# “Being in Control of My Asthma Myself” Patient Experience of Asthma Management: A Qualitative Interpretive Description

**DOI:** 10.3390/pharmacy6040121

**Published:** 2018-11-15

**Authors:** Damilola T. Olufemi-Yusuf, Sophie Beaudoin Gabriel, Tatiana Makhinova, Lisa M. Guirguis

**Affiliations:** 1 Faculty of Pharmacy and Pharmaceutical Sciences, University of Alberta, Edmonton, AB T6G 1C9, Canada; damiade@ualberta.ca (D.T.O.-Y.); sophie.beaudoin@gmail.com (S.B.G.); tatiana.makhinova@ualberta.ca (T.M.); 2 Asthma Working Group of the Respiratory Health Strategic Clinical Network, Alberta Health Services, Calgary, AB T2W 1S7, Canada

**Keywords:** asthma, patient experience, patient-centred care, communication, patient education, patient-pharmacist relationship, self-regulation, qualitative interpretive description

## Abstract

Asthma control can be achieved with effective and safe medication use; however, many patients are not controlled. Patients’ perceptions of asthma, asthma treatment, and pharmacist roles can impact patient outcomes. The purpose of this study was to explore patients’ experiences and patient–pharmacist relationships in asthma care. Qualitative Interpretive Description method guided the study. Semi-structured individual interviews were conducted with 11 patients recruited from personal contacts, pharmacies, and asthma clinics. Categories and themes were identified using inductive constant comparison. Themes indicated patients had a personalized common sense approach to asthma management, “go-to” health care provider, and prioritized patient–pharmacist relationships. Patients described their illness experiences and asthma control based on personal markers similar to the common sense model of self-regulation. Patients chose a family physician, asthma specialist, respiratory therapist, or pharmacist as an expert resource for asthma management. Patient perceived pharmacists’ roles as information provider, adviser, or care provider. Pharmacists who develop a collaborative relationship with their asthma patients are better positioned to provide tailored education and self-management support. Inviting patients to share their perspective could increase patient engagement and uptake of personalised asthma action plans to achieve asthma control.

## 1. Introduction

Asthma is a significant public health problem all over the world and an everyday reality for the 2.4 million Canadians living with asthma [1]. Asthma control can be achieved with effective and safe medications and treatment guidelines [2]. However, level of asthma control has not improved over the last decade and currently 9 out of 10 Canadians with asthma are out of control [3]. Poor asthma control is burdensome to patients and increases emergency room visits, hospitalizations, and absence from work or school [4].

The reasons for poor asthma management are multifaceted including the disease itself, presence of comorbidities, patients’ self-management, healthcare professionals’ care, or the interaction among these factors [5,6]. Medication therapy is the primary intervention used in asthma highlighting asthma patients have needs that pharmacists can address [7,8]. Pharmacists frequently encounter asthma patients and are not only well placed to identify patients with poorly controlled asthma but also resolve medication problems, educate patients on inhaler technique, monitor therapy, and develop personalised asthma action plans [8,9,10]. 

Pharmacist-delivered interventions in asthma management have improved patient outcomes [11,12,13,14,15] but only few have explored the patient experience of pharmacist care as one of the factors contributing to positive outcomes [16,17,18]. Understanding the patient, their beliefs and experience about asthma and asthma treatment reduces barriers to effective asthma treatment [19,20]. Patients involved in their care are more likely to communicate their goals, preferences, and concerns, to seek support in adhering to their care plan, and to take more ownership of their treatment [20,21,22]. More so, effective communication between patients and pharmacists provides an enabling context for optimizing therapy and achieving asthma control for patients [17,23,24]. The patient’s experience in managing asthma and how they are supported by pharmacists needs to be explored further. 

With the shift toward patient-centred care in Canada and around the world, pharmacists have taken on an expanded role in providing patient care services to those living with chronic conditions including asthma [25]. In the province of Alberta, where the scope of pharmacy practice is the widest (pharmacists can prescribe for minor ailments, renew, adjust, initiate or substitute prescriptions, administer vaccinations, order and interpret laboratory tests, conduct medication reviews, and develop care plans), the experiences of those living with asthma are important in order to enhance care [26]. Given that the patient’s perspective has not been examined in this patient-focused practice model, we sought to understand how asthma patients have experienced pharmacists’ care. Our findings could have potential application in the design of patient-centred interventions in pharmacy care and improve care for asthma patients. Our study objectives were:

1. To identify how patients manage their asthma
2. To describe what resources patients need to access for asthma management
3. To understand how patients view and experience pharmacists’ roles in ongoing management of asthma.

## 2. Methods

### 2.1. Research Team and Reflexivity

The research team consisted of three researchers, one patient with asthma (i.e., only research team member with asthma), and one pharmacy student, who were all invested in improving the quality of care for people with asthma. All three researchers were pharmacists; however, none was practicing. The pharmacy student conducted the interviews and was trained and supervised by one of the authors (L.G.). The patient was identified as a patient adviser with the Alberta Health Services Respiratory Health Strategic Clinical Network (RSCN) where two researchers were active members. The patient participated in one interview and reviewed a subset of interviews to provide insights on patient perspectives.

### 2.2. Study Design

Interpretive Description was the qualitative methodology used to frame the study [27,28]. Its constructivist paradigm formed the basis of our theoretical approach to knowing how patients perceived and interpreted meaning created through their asthma experiences [27,28]. The constructivist position recognizes that multiple perspectives exist and fit with our study objective of understanding the different perspectives of asthma patients’ self-management in community pharmacy practice. As an applied qualitative approach, interpretive description focuses on thoughtful consideration of factors that could influence practice such as practice models, professional mandates, and biases of individuals and disciplines [29,30]. The orientation toward practice settings ensures that the creation of knowledge that is relevant to real-world clinical practice as well as theory development or expansion. 

We used an inductive approach without the influence of a priori theories though we were open to theory to inform the analysis and discussion. We favoured the use of theory as an analytical tool rather than as a theoretical framework made explicit from the beginning to allow for the generation and interpretation of new themes that fit into broader contexts [31,32]. This study was driven by the need to better understand the experiences and perspectives of patients on managing their asthma and the role of community pharmacists in order to generate knowledge that could improve care practices for asthma patients. Ethics approval for the study was granted by the University of Alberta Health Research Ethics Board (study ID Pro00065978) prior to recruitment.

### 2.3. Recruitment and Sample

Adults between 18 and 70 years old were eligible if they or their children had asthma, took at least one asthma medication, spoke English, and were able to consent. Patients were ineligible if they had limited capacity to communicate. Purposive and convenience sampling were used to recruit 11 patient participants from three settings. The first five patients (i.e., Patients 1–5) were recruited from personal networks of members of the RHSCN. It was noted that these patients had positive experiences with pharmacists and thus we purposively sample two patients (Patients 6 and 7) from a community pharmacy with a known high level of care. Finally, four patients were purposively recruited from an asthma clinic to capture the voices of patients who may have more complex or severe asthma (Patients 8–11) than those in primary health care sites. 

### 2.4. Data Collection

Data collection consisted of semi-structured individual interviews conducted by the trained pharmacy student who did not know the participants. Interviews occurred face to face and interviews occurred by telephone as participants lived out of Edmonton or they preferred to do so for scheduling or personal reasons. Seven interviews were conducted between July and August 2016. Additional four patients were recruited and interviewed in June 2017. Interviews were guided by a semi-structured interview guide lasted between 16 and 38 min. The questions in the interview guide were developed based on literature and knowledge of asthma care and community pharmacy practice (Appendix A). We explored the patient’s experience of asthma, management strategies, and interactions with community pharmacists. As data collection and analysis occurred concurrently, areas of emerging interest were noted and included as probes in the next interview. Thus, the interview guide was modified as the interviews progressed to reflect additional questions.

During and after each interview, handwritten field notes were taken to record important observations made at the interview such as non-verbal behaviour, interpersonal disposition, and the researcher’s impressions of the participant within the context of the interview. All interviews were audio recorded and transcribed verbatim.

### 2.5. Data Analysis

Interpretive description methods and thematic analysis shaped the data analysis [27,28,33,34]. First, researchers wrote a summary of each patient interview, included information about each patient and potential themes. Each transcript was open coded to identify main ideas and develop a taxonomy of related codes. Similar codes were sorted and collapsed together to create categories. Categories were then compared and contrasted across transcripts to generate themes that ran through the data. Constant comparison was used to compare between patients, provider preferences, type of pharmacist relationships to refine themes. Themes moved beyond clustering similar categories to develop a conceptual understanding of the patients’ experiences with asthma [33,34]. We also used theory to create themes which contributed to making sense of the data and contextualizing study findings within the body of knowledge. Without the use of theory and thematic analysis, the results of the research will have little meaning and application in real world practice [31,35,36]. We involved one patient participant in the data analysis to draw on her patient experiences in the interpretation of a subset of interview transcripts. This helped improve the study’s transparency and trustworthiness. 

### 2.6. Rigour

Rigour was ensured throughout the study. First, the study was designed to achieve congruence between the research question and the theoretical position. Recruitment was adapted to capture patients with varying levels of asthma control and types of pharmacist relationships. Data collection and analysis were conducted concurrently to ensure our ongoing interview explored both new and evolving ideas introduced by participants. Analysis involved interview summaries including field notes, four coders, and iterative coding process. The recognition of three types of patient–pharmacist relationships allowed for multi-faceted comparisons between patients. A patient who was interviewed also participated in the data analysis to bring her experiences and improve the study’s transparency and trustworthiness. 

## 3. Results

The sample was predominantly female (n = 8, 73%) with a mean age of 42 years (range = 24 to 56 years). Patients recruited by the members of the RHSCN formed the largest group followed by the asthma clinics and then the community pharmacy. Within two groups sampled from the RSCN and asthma clinics, we observed variation in patients’ experiences of interacting with pharmacists. Four patients visited family physician clinics, another four saw an asthma specialist, and one patient used a respiratory therapist for routine asthma care. One patient used both a respiratory a family physician and pharmacist and another an asthma specialist and pharmacist. The characteristics of patients are shown in Table 1.

### 3.1. Theme 1: Personalized Common Sense Approach to Asthma Management

The patients described their illness experiences in a personal way. Similar to Leventhal’s Common sense model of self-regulation [37], their perceptions of asthma symptoms, level of control and emotions were modifiable based on their knowledge and feelings of what they experienced over time. Patients discussed how they made sense of asthma symptoms, came up with their own individual non-medical strategies for coping and monitoring improvements or worsening of symptoms. What was most striking is that the patients constructed representations of their asthma from their everyday experiences but these narratives were not always discussed in medical visits. Patient ideas were considered non-medical and diverged from biomedical conversations that predominantly occur in medical settings. 

#### 3.1.1. Sub-Theme 1: Personalized Markers for Self-Monitoring

Patients determined if their asthma was under control based on personal markers of control and severity. Patients regularly made connections between symptoms and severity of their condition, relying on these subjective experiences as an early warning of an impending attack. Here are three individual examples of patient symptoms:

As soon as I start coughing, I know this is going to be a three-month adventure, trying to figure out why.

*(Patient 1)*

So as soon as I start to get a tickle in my throat, and kind of that feeling that there’s feathers in there…then I make sure that I take my Advair^®^ at night time as well,

*(Patient 6)*

I know if I can’t make it up the stairs without wheezing, or a problem with my breath, that’s when I go to my primary care doc.

*(Patient 3)*

After recognizing the seriousness of the condition, patients knew what steps to take to address symptoms. Patients’ self-management practices combined both medical (e.g., mainly medications) and non-medical lifestyle strategies to control current symptoms and prevent future problems.

#### 3.1.2. Sub-Theme 2: Personalized Non-Medical Measures

Mostly, non-medication related strategies were seen to be as beneficial as medications for day to day management. One patient took preventative measures.

So probably lifestyle wise is my biggest thing—like, you know, I try and exercise, get my cardiovascular as much as I can, make sure that my core is strong and strengthen everything around there.

*(Patient 4)*

Other patients had non-medication strategies for asthma attacks

What I used to do was just to try to lay [sic] down, relax my muscles—there are a lot of times when you have an asthma attack, your core muscles are kind of working overtime to force air in and out of your lungs. Um, so, just the more relaxed I can make myself, the better.

*(Patient 2)*

#### 3.1.3. Sub-Theme 3: Personalized Access to Medication

Patients figured ways to obtain cheaper medications and avoid the hikes in travel health insurance premiums that result from asthma attacks and their association use of oral emergency medications. With insights into the ease of getting medications in less regulated markets outside of Canada (e.g., Mexico), Patient 3 and 9 were sensible to proactively stock up on asthma medications to ensure they always had sufficient supply to meet asthma needs. It was clear that these patients knew what they needed and actively pursued practical solutions.

Now, the fun part about prednisone is, for some insurance companies, it’s an indicator of disease severity—especially if you’ve taken it in the last six months and all that, and they won’t insure you for travel. So, like I said, I handle my asthma, and I know when I need to hit it hard and hit it quick. And, so, what you end up doing too is you have some patients—such as maybe myself—going down to Mexico, getting prednisone. Because they know that, number one, it’s an insurance flag if you take it.

*(Patient 3)*

### 3.2. Theme 2: Patients Identify Their “Go-To” Health Care Provider

Although patients apply a common sense view to manage and monitor asthma symptoms, the chronic nature of asthma implies frequent contact with the health care system. In our study, all patients used their common sense approach to choose one or more healthcare providers for routine asthma care. This “go-to” person was a family doctor, asthma specialist, respiratory therapist or pharmacist whom they recognized as an expert resource for asthma management. The patients described benefits of the encounter such as prescribing new medication or refills, a demonstration of proper inhaler technique, monitoring asthma medications.

Would I have booked an appointment to come in, and sit down, and do this [asthma action plan] with them? Probably not. I think that’s probably—you know, that’s something again that I would reserve for my family doctor, but, (I) would appreciate that.

*(Patient 2)*

When I go to [name of Asthma specialist] for the respiratory end of things, she’ll ask if I need any prescriptions.

*(Patient 9)*

The choice of provider was informed by their perceived needs and expertise of the healthcare provider. Four patients saw their family physician (Patients 1, 2, 3, and 8) as the go-to-healthcare provider. Patients 4, 9, 10, and 11 that did not trust their family physician to provide expert advice on asthma preferred to seek help from an asthma specialist who focuses on asthma patients. Another patient (Patient 5) saw a respiratory therapist. Patients 6 and 7 had positive relationships with their pharmacist thus listed the pharmacists and either their family physician or asthma specialist respectively. The preferred provider(s) for each patient is outlined in Table 1.

### 3.3. Theme 3: Patient–Pharmacist Relationship Comes First

**Figure 1:**
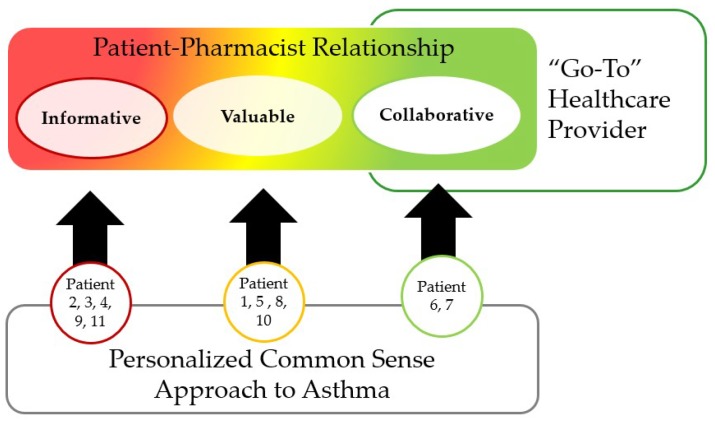
Relation of Spectrum of Patient–Pharmacist Relationships to Study Themes. Pharmacists were only considered the go-to healthcare providers when there was a collaborative relationship.

Patient and pharmacist relationships ranged from non-existent to an ideal situation where the pharmacist partnered with the patient. These relationships influenced care and how their asthma needs were met (Figure 1). For instance, Patients 6 and 7 had the only collaborative relationship with their pharmacist and correspondingly were the sole patients to consider their pharmacist a “go-to” provider. Patient 4 aptly described how relationships are important to patient care.

I think it takes some time to build trust, that sometimes is built in that patient-physician, that’s important… But if you’re comfortable, I think a pharmacist is well within their scope of practice to help create an action plan, for sure.

*(Patient 4)*

#### 3.3.1. Sub-Theme 1: Information-Focused Relationship

Majority of the patient–pharmacist relationships clustered toward the bottom end of the spectrum where there is little existing positive relationship or a negative relationship. A group of patients described pharmacist interactions as “impersonal” and transactional. In such cases, they felt the relationship with the pharmacist was limited to dispensing without actively engaging the person behind the prescription.

It’s [the interaction] very brief. It’s just because I’ve had it for so long, that they know that I know what I’m doing with it. Um, but yeah, there’s no real discussion regarding the medication.

*(Patient 2)*

Generally, patients seemed to have low expectations of the pharmacist because they were satisfied with brief straightforward conversations and minimal interaction with the pharmacist.

The challenge is, it’s [role of pharmacist] only appropriate in what’s asked, right—I mean, nobody really wants anything forced on them, so, um… I imagine a pharmacist who is a certified respiratory educator would be a great thing, and [inaudible] lean on that background and work with them… But essentially in the end, I just want to know about the drug, interactions, possible side effects, and how to do it properly.

*(Patient 4)*

Similar experiences evoked different reactions in patients. One patient explained that being disappointed by their pharmacist’s inability to renew prescriptions for asthma medications in an emergency caused expectations to drop very low.

Didn’t engage me on, yeah, “How well is he controlled? What is he doing about it? Does he know what he’s doing?” Right? I rarely get that question. But once again, I think that’s because in the last few years, I just… I just see that pharmacy as a dispensing outlet.

*(Patient 3)*

The type of patient–pharmacist relationship can be also understood from the perspective of trust. People may have an inherent distrust in the involvement of pharmacists in the health care system. Those who hold this view believe that any advice beyond filling a prescription and drug interaction information is unsolicited and out of professional boundaries. Our study revealed one patient with such view. The patient opposed pharmacists monitoring asthma drug therapy, developing an asthma action plan and preferred to stick to her asthma specialist who has the specialised knowledge and expertise to assess and manage asthma.

“Well it’s a nightmare to be honest with you. I find that whenever I’ve gone to a pharmacy in [city name] I have either had a pharmacist try to tell me what my asthma medication should be or they try to ask you tons of questions because you have asthma, but they don’t know the whole picture. They only see the prescription that you’re bringing in, they don’t have your medical file, they don’t know how many doctors you’ve been to or what steps you’ve taken so far. They just assume that because you have asthma, it’s not under control and they can provide additional advice that is actually unsolicited. It’s really irritating to be honest with you”.

*(Patient 11)*

#### 3.3.2. Sub-Theme 2: Valuable Relationship

The middle group of patients had a relationship with their pharmacists where the pharmacist knew them by name and supported technical skills by demonstrating inhaler technique. These patients attested to the value of pharmacist-provided patient education since many patients have poor inhaler technique. For example, one patient alluded to the fact that using the Aerosol holding chamber recommended by a pharmacist eased taking her rescue medication and improved its effectiveness.

I do [use the AeroChamber^®^] yeah. Yeah I don’t even see that as a concern because I’ve always used it. It was actually a pharmacist who told me about that, not my family doctor. Which is part of why I wanted to participate in this because they play such an important role and that’s made such a difference for me.

*(Patient 8)*

Patients in this category demonstrated curiosity and desired additional care that might improve their asthma condition beyond filling prescriptions. They wanted to ask questions on medication effectiveness, user-friendly devices, and latest advancements in asthma therapy. Patients hoped to involve the pharmacist in their care to a greater extent but felt the pharmacists was responsible for initiating conversations and creating more awareness of patient care services. Though the patients have not experienced pharmacist prescribing or care plans, they indicated these would be helpful if done in collaboration with their family physician who has more knowledge of the patient’s medical history.

Or maybe even like when I get to a pharmacist, having an appointment with them when I go to pick it up, to go over things and maybe decide if this even going to help me, instead of spending three months trying to figure it out with my doctor, who maybe not as knowledgeable on that kind of stuff. So yeah, it’d be nice—I mean, I know they offer that kind of stuff, so maybe making it more known

*(Patient 1)*

Reflecting on previous challenges in obtaining medications, some patients gave suggestions on ways to enhance access to pharmacy services. For example, relocating the pharmacy to the front of the building would facilitate access for urgent medication pickups. Additionally, pharmacists should have the mandate to refill medications for patients with controlled asthma in order to free up time for doctors to attend to sicker patients with greater medical needs.

I understand that there might be insurance stuff involved in that but I do believe that I should not have to make an appointment with my doctor just to get medication refills. I think that is ridiculous and it’s a waste [of time]. There is legitimate sick people out there that really need to see the doctor and all that does is put stress on an already stressful system. So I really think the doctor should just be able to push a button to refill the prescriptions at the pharmacist or something. The pharmacy should be able to refill your medication without your doctor.

*(Patient 10)*

#### 3.3.3. Sub-Theme 3: Collaborative Relationship

Two Patients (6 and 7) credited their ability to take control of their asthma to the written asthma action plan co-created with their pharmacist. They visited the same pharmacist who is a Certified Asthma Educator (CAE) and spoke very highly of the pharmacist’s commitment and advocacy in helping them become empowered in managing their asthma and monitoring the effectiveness of medications.

Her [the CAE pharmacist’s] role is to advocate for being in control of my asthma myself, and making sure that the things she or my doctor are prescribing are actually benefitting me in the way they’re supposed to, because I have the right knowledge to use them properly”.

*(Patient 6)*

These two patients valued the one-to-one consultation that could have with the pharmacist saying that these were helpful in reviewing and reinforcing the patients’ knowledge of asthma and monitoring asthma goals. One patient specifically said that her pharmacist keeps encouraging her to be symptom-free and that goal motivates her to know all aspects of her condition and maintain control of symptoms.

You know, she’s really, really educated me on all aspects of asthma—it’s been a real eye opener. Because I’ve had it for so long, and for probably at least the first fifteen years or so, I was just… Kind of on my own. You know, I’d go to the doctor. “Oh hi, how you doing?” “Um yeah, kind of coughing.” You know, he was pretty good, but he didn’t help me understand why I was coughing, and why I was having these symptoms. Whereas she sits me down and says, “Okay, look. This is why you’re doing this, this, and this. And this is what you need to do. And you should not cough, period.” [laugh].

*(Patient 7)*

Apart from providing a better understanding of the illness and medications, the patients acknowledged their relationship with the pharmacist was immensely supportive. One patient in particular worried and felt she had to tough it out on her own before having an action plan. Now she felt supported to change her medication-taking behaviour and successfully monitor symptoms. Being able to know when control is worsening and what specific actions to take to tackle asthma exacerbations were important benefits of the asthma action plan for these patients.

And I’ve always wanted one. I would ask the doctor, and… They either didn’t know about it or didn’t have time. But she [the pharmacist] will sit down with me every month or two and go through it and do any changes…. So the action plan gives me specific things to do when my chest gets tight, when I get short of breath—then I can be flexible with the Symbicort. And I don’t have to be afraid to take more Ventolin, or take even less than, like, one an hour. If it gets bad, I can do more.

*(Patient 7)*

The pharmacist was described as being non-judgmental, caring, personable, attentive, and listening deeply to understand the patient’s situation and encourage them toward their asthma goals. The two patients who had a positive and trusting relationship with their pharmacist have experienced comprehensive asthma care.

[Speaking about pharmacist] “and be very caring and personable during the process”

*(Patient 6)*

And [the pharmacist] is so good that way, she’s always encouraging me to, you know—by the time the next action plan comes up, ‘Oh yeah, I was supposed to do that, okay’. So she doesn’t judge, she doesn’t criticize, she just (says), ‘Okay, well let’s work on that’.

*(Patient 7)*

## 4. Discussion

This study extends the knowledge in pharmacy literature by exploring patients’ experiences of asthma within the movement toward patient-centred care in pharmacy practice. Previous studies that examined pharmacists’ contribution to asthma care found that pharmacists possess the skills and competencies required to support patients with self-management education, demonstrating inhaler technique, and monitoring asthma therapy and could do more for patients in therapy monitoring and follow-up [7,8,38]. Findings from prior studies highlight the need for community pharmacists to work together with patients to attain control of their asthma. This study adds to earlier work by privileging the patient’s perspectives on how they experienced the community pharmacist’s delivery of asthma care. We found three themes namely: patients had a personalized common sense approach to asthma management, had a “go-to” health care provider to address asthma needs, and considered patient–pharmacist relationships important in asthma care (Figure 1).

Patients mostly evaluate their level of asthma control based on personally defined parameters and represent and manage asthma in a way that is consistent with the common sense model of self-regulation [37]. This model describes how patients rely on a mentally constructed approach to assign meaning to their illness symptoms (or lack thereof) and how this affects care seeking and self-management behaviours. Previous studies have demonstrated the relationships between asthma patients’ representation of illness and medication adherence [21,39]. Our study adds to prior knowledge that caring for asthma patients goes beyond understanding the disease, clinical presentation, or providing medications to understanding the patient. The study points to the need to develop a shared understanding with the person living with asthma including their perceptions of asthma and asthma treatment as well as shared goals of asthma control [19,40]. Knowing that many patients’ representations are dynamic and based on experiences, pharmacists, and other healthcare professionals could employ patient-centred communication [41,42]. Patient-centred communication involves inviting the patient to share their perspective, addressing concerns about treatment, regularly monitoring asthma medications and level of control in individual patients [20,43,44]. Patients knowledge and experiences should be recognized as valid during routine encounters to make interactions more meaningful and patient-focused [45].

In addition to adopting personal definitions of asthma, patients with asthma drew on a variety of resources within the healthcare system to manage their condition [46]. This was also reflected in the range of preferred healthcare providers for patients in our study. This “go-to” person included a family physician, asthma specialist, respiratory therapist, or pharmacist, or at least two of these providers, who they recognized as an expert resource for asthma information, advice and support. The nature and development of relationships with the health care provider is influenced by the perceived asthma needs and perceived role of the health care provider as being able to provide good asthma care, level of trust, convenience, and ease of access [19]. It may be that patients who had at least two “go-to” health care providers may have had complex needs or reasoned that they need more than one healthcare professional to address their needs. While it is desirable for asthma specialists to care for people with complex asthma, they are neither sufficient in number nor accessible to patients when needed. More so, patients with chronic obstructive pulmonary disease receiving both care specialist and primary care were not shown to have better outcomes than those receiving primary care only, though the case for asthma may be different [47]. Patients mentioned the benefits of having a regular health care provider was to prescribe new medication or refills, check and demonstrate proper inhaler technique, monitor asthma medications. Patient can access these services in a community pharmacy in Alberta where a government funded model supports all pharmacists to extend prescriptions and provide care plans as well as approved pharmacists to initiate new medications [48]. The reliance of patients on different healthcare providers would be an important factor to consider if a shift to multidisciplinary team care delivery were to occur. Another factor would be if patients’ choice of healthcare provider changes with time though our study did not examine this possibility.

Patients’ expectations of pharmacist roles and the type of patient-pharmacist relationship influences perceptions on the quality of care and this was supported by our findings [49,50]. Majority of the patient-pharmacist relationships clustered toward the bottom end of the spectrum where there is little positive relationship or even a negative relationship. On the other end, two patients had positive relationships with their pharmacists that impacted their asthma care. Our study conceptualises patient–pharmacist relationship (Figure 1) as a continuum of information-focused, valuable, and collaborative relationships similar to patient’s perceptions of pharmacist roles as retailers, medication experts, and care providers [49,50,51,52,53,54,55,56,57]. While policy changes to enhance care for people with chronic conditions has been a significant driver of pharmacists expanded role, every patient encounter is an opportunity to build the relationship and support the uptake of patient care services [58]. The use of patient-centred communication strategies are linked to higher therapy monitoring and the use of asthma action plans, an important self-management tool [23]. Compared with patients with a low engagement with pharmacists, those who had valuable and collaborative relationships reported they not only had better understanding of asthma and asthma treatment but also more confidence and skill in managing their asthma. 

The themes in our study reflect that asthma patients value patient-centred communication in their encounter with pharmacists which has the potential to increase patient acceptance of professional roles [59,60]. This implies pharmacists could invite patients to share their perspective asthma and common sense approaches to achieving control. This could increase patient engagement and tailoring of asthma education focused on patient needs. The practice of pharmacy has expanded beyond traditional dispensing roles to a more collaborative relationship and pharmacists should become competent in incorporating specific markers of asthma control into personalized asthma action plans. What matters to patients in their asthma management is just as important as telling patients what to monitor.

### Limitations

Our study focused on patients’ perspectives only which are different from providers’ perspectives [19]. Future work could compare the experiences of patients and pharmacists to provide a complete understanding on the dynamics of the patient–pharmacist interaction and how it influences the quality of asthma care. Another limitation was that all the patients were experienced, had an asthma diagnosis for a long period of time and mostly likely learned how to navigate the health system and adequately manage their asthma. Since their views may be different from newly diagnosed patients where pharmacists have been known to spend more time with patients, more research is needed to explore the initial interactions between new asthma patients and pharmacist in the era of higher public and patient interest in pharmacist roles. Although the patient and pharmacist relationships have been the focus in the patient-centred care, a broader range of “system” factors may affect the patient experience care but our study did not investigate how structural and political factors influence patient attitudes and experience of asthma care [61]. Further research is necessary to evaluate strategies at the organization and policy level that could foster the patient-centredness of care within pharmacy practice. 

## 5. Conclusions

Patients had a personalized common sense approach to asthma management, a “go-to” health care provider to address asthma needs and considered patient-pharmacist relationships important in asthma care. Our study indicated that asthma patients viewed pharmacists as retailers, medication experts, or care providers and their perceptions were shaped by their beliefs and experience of the pharmacist role. Pharmacists routinely encounter asthma patients and would be better positioned to provide tailored education and self-management support if they developed a collaborative relationship with patients and invited patients to share their common sense approaches to asthma. This starting point could increase patient engagement and uptake of personalised asthma action plans to achieve asthma control.

## Figures and Tables

**Table 1:** Patients’ demographics and provider relationships associated with asthma care.

Patient Identifier	Age	Gender	Recruitment Strategy	“Go-To” Healthcare Provider	Type of Pharmacist Relationship
Patient 1	24	F	RSCN	Family physician	Valuable
Patient 2	43	M	RSCN	Family physician	Information
Patient 3	41	M	RSCN	Family physician	Information
Patient 4	51	M	RSCN	Asthma specialist	Information
Patient 5 ^a^	45, 18, 14	F, M, M	RSCN	Respiratory therapist	Valuable
Patient 6 ^b^	54	F	Community Pharmacy	Pharmacist and Family physician	Collaboration
Patient 7 ^b^	56	F	Community Pharmacy	Pharmacist and Asthma specialist	Collaboration
Patient 8	30	F	Asthma Clinic	Family physician	Valuable
Patient 9	56	F	Asthma Clinic	Asthma specialist	Information
Patient 10	30	F	Asthma Clinic	Asthma specialist	Information
Patient 11	36	F	Asthma Clinic	Asthma specialist	Information

^a^ Patient has two teenage sons who have asthma; ^b^ Patients have the same pharmacist who is a Certified Asthma Educator.

## Appendix A. Patient Interview Guide

Demographic information

1. How old are you? _______
2. Gender: __Male __Female
3. How long have you had asthma?
4. What medications do you use for your asthma?

Part 1: Asthma

1. Tell me about how you manage asthma. Who helps you with that?
2. Tell me about your asthma medications
3. Tell me about how you manage your asthma when you have a flare up/out of control?
4. When thinking about your asthma and your life, what matters to you?
5. Do you use an asthma action plan? (Show a copy). If so, please tell me how it was created?
6. Who helps you manage your asthma?
  - Family Physician, Asthma Clinic, Asthma Specialist, Nurse, Pharmacist?

Part 2: Pharmacist Care

1. Tell me about a typical interaction with your pharmacist about your asthma medications.
2. What is the pharmacist role in the interaction that occurs between pharmacists and patients?
  - Has a pharmacist prescribed an inhaler for you? How did that happen?
  - Have you had a med review or care plan session with your pharmacist? Has a pharmacist had a longer talk with you that concluded with signing a piece of paper? Please tell me about it.
3. What is your role in the interaction that occurs between pharmacists and patients?
4. Imagine that your pharmacist wants to do more for you. What could your pharmacist do to help with your asthma?
5. Tell me about any negative experiences you have in the pharmacy.
6. What would you think of creating an Asthma Action Plan with your pharmacist?
